# Prise en charge conservatrice des hématomes puerpéraux de gros volume: à propos de 3 cas

**DOI:** 10.11604/pamj.2013.16.9.1918

**Published:** 2013-09-12

**Authors:** Mehdi Kehila, Sonia Ben Khedher, Dorra Zeghal, Sami Mahjoub

**Affiliations:** 1 Service C, Centre de maternité et de néonatalogie de Tunis, Tunisie

**Keywords:** hématome puerpéral, postpartum, traitement, hémorragie, puerperal hematoma, postpartum, treatment, hemorrhage

## Abstract

L'hématome puerpéral est une complication rare parfois grave du post-partum. Sa prise en charge reste encore non consensuelle. L'objectif de notre travail est de discuter la possibilité et l'intérêt d'une prise en charge conservatrice en cas d'hématome de gros volume sous couvert de certaines conditions. Ainsi nous rapportons 3 cas d'hématomes péri- génitaux de taille supérieure à 8cm pour lesquels un traitement conservateur a été préconisé. L'évolution était favorable pour les 3 patientes.

## Introduction

L'hématome puerpéral ou thrombus péri-génital est une complication hémorragique rare et potentiellement grave du post-partum qui demeure mal connue [1]. Cette pathologie peut mettre en jeu le pronostic vital maternel [2]. Faute de données suffisantes dans la littérature, la conduite à tenir est loin d'être uniforme. Le traitement est fonction de la taille de l'hématome, de la disponibilité de la radiologie interventionnelle et plus encore des habitudes des équipes obstétricales [3]. La prise en charge des hématomes de grande taille reste le sujet le moins consensuel. En effet, la plupart des auteurs sont pour une intervention systématique par embolisation ou chirurgie dans ce cas. Nous rapportons 3 cas d'hématomes génitaux de gros volumes (>8cm) traités de façon conservatrice (Tableau 1). Nous discutons l'intérêt de cette prise en charge et les conditions dans lesquelles nous préconisons ce type de conduite.

**Tableau 1:** Tableau récapitulatif des 3 observations

	Age (Ans)	Gestité/parité	Mode d'accouchement	Poids fætal	Diagnostic positif	Siège et taille de l'hématome	Biologie	Conduite à tenir	Evolution
*Patiente 1*	36	G1 /P1	AVB	3300g	-H2 de post-partum -Douleurs périnéales	Bématome de la paroi vaginale gauche de 12cm	Hb = 10 TP = 100%	Vessie glace AINS antalgiques	Disparition de l'hématome au bout de 8 semaines
*Patiente 2*	42	G1/P1	AVB	3400g	-J5 de post-partum -douleurs périnéales -dysurie	Bombement de la face latérale droite du vagin de 12cm	Hb = 7,2 TP = 90%	Tampon vaginal (24h) Transfusions AINS antalgiques	Disparition des douleurs au bout de 15j disparition de l'hématome au bout de 40j
*Patiente 3*	28	G1/P1	Forceps	3500g	-H3 de post-partum -douleurs hypogastriques - TV: utérus ascensionné latéro dévié à gauche + douleur du cul de sac vaginal droit	Hématome du ligament large de 10 cm	Hb = 9 TP = 100%	Transfusions AINS antalgiques	Disparition de l'hématome au bout de 6 semaines

AVB: Accouchement par voir basse

## Patients et observations

### Observation 1

Il s′agit d′une patiente âgée de 36 ans, deuxième pare, ayant accouché par voie basse avec épisiotomie d'un garçon pesant 3300g. Deux heures après l'accouchement, on a découvert à l'inspection une tuméfaction vulvo-périnéale du coté controlatéral à l'épisiotomie faisant 12 cm de grand axe. Les constantes hémodynamiques étaient stables et la biologie était correcte (Hb = 10g, TP = 100%). Devant le caractère la stabilité de la taille de cet hématome à la surveillance, l'absence de douleur insupportables et l'état hémodynamique stable, on a opté pour un traitement médical conservateur (vessie de glace + AINS). L'évolution était favorable avec une diminution progressive de la taille de l'hématome jusqu'à disparition totale à 8 semaines post-partum.

### Observation 2

**Figure 1:**
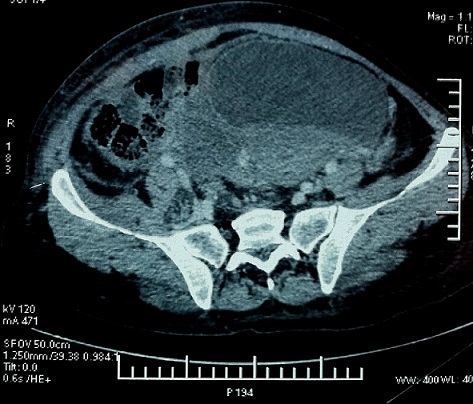
Hématome latéro-utérin droit mesurant 13x6 cm dans le plan axial et étendu sur 13 cm en hauteur

Il s'agit d'une patiente âgée de 42 ans primipare ayant accouché par voie basse avec épisiotomie d'un bébé de sexe féminin de poids de naissance 3400g. La patiente était mise sortante à J2 de post partum, l'examen à la sortie était sans particularité. La patiente a été ré hospitalisée à j5 de post-partum pour des douleurs périnéales ayant débuté le soir de sa sortie et qui ont augmenté d'intensité progressivement avec apparition secondaire d'une dysurie. A l'admission, la patiente était stable sur le plan hémodynamique, le toucher vaginal et l'examen sous valve ont révélé un bombement de la face antérieure et latérale droite du vagin de 10 cm de grand axe. La biologie a montré une anémie à 7,2 g/dl et un bilan d'hémostase correcte. Un tamponnement vaginal a été réalisé par la mise d'un champ tétra dans le vagin après administration de morphine et la patiente a été transfusée par 4 culots globulaires. Le taux d'hémoglobine après transfusion a demeuré stable à 10,5g/dl sur 2 NFS à 12h d'intervalle. Devant la stabilité clinique et biologique un scanner pelvien a pu être réalisé et a objectivé un hématome péri-vaginal antérieur fusant en latéro-utérin droit et faisant 13 cm de grand axe (Figure 1). Devant la stabilité hémodynamique et biologique et après concertation avec les radiologues interventionnels nous avons opté pour une simple surveillance (hémodynamique et biologique) associée à un traitement symptomatique par antalgiques et anti-inflammatoires. Le tampon vaginal a été enlevé au bout de 24h. La patiente est demeurée stable à la surveillance, elle a été mise sortant à j 5 d'hospitalisation. La dysurie a disparu 5 jours après la sortie de la patiente, les douleurs se sont améliorées rapidement pour disparaitre totalement au bout de 15 jours et l'hématome a disparu au toucher vaginal au bout de 40 jours.

### Observation 3

Il s'agit d'une patiente âgée de 28 ans primipare, ayant accouché par forceps avec épisiotomie d'un nouveau-né pesant 3500g. A H3 de post-partum la patiente a présenté une douleur hypogastrique résistante aux antalgiques de palier 1. L'état hémodynamique de la patiente était stable. L'examen clinique a montré une sensibilité hypogastrique, un bon globe utérin et l'absence d'un saignement extériorisé. Le toucher vaginal a révélé un utérus ascensionné latéro-dévié à gauche avec une douleur et un bombement au niveau du cul de sac vaginal droit. La patiente était tachycarde à 100 battement/min avec une TA correcte à 12/8. Une échographie pelvienne pratiquée a montré la présence d'une image latéro-utérine droite de 10 cm évoquant en premier lieu un hématome de ligament large. Une NFS réalisée en urgence a montré une anémie à 9 g/dl (la patiente partait de 11,2 g/dl avant l'accouchement). Devant la non disponibilité de l'embolisation radiologique pendant les gardes dans notre pratique, il a été décidé de commencer la transfusion sanguine et d'intervenir chirurgicalement si aggravation de l'état hémodynamique ou si déglobulisation rapide. L'évolution immédiate a été marquée par une stabilité hémodynamique et une augmentation du taux de l'hémoglobine à 11 g/dl après transfusion de 2 culots globulaires. A l'échographie de contrôle après 4 heures l'hématome paraissait stable. On a opté pour un traitement médical conservateur (AINS, antibiotiques) associé une surveillance clinique stricte en milieu de réanimation obstétricale à proximité immédiate du bloc opératoire pendant les 24 premières heures. L'évolution immédiate était favorable avec persistance d'une stabilité hémodynamique, biologique puis par la suite régression progressive de la taille de l'hématome jusqu'à disparition complète échographique au bout de 6 semaines.

## Discussion

L'hématome péri-génital ou puerpéral est une forme particulière, rare, de l'hémorragie du post-partum pouvant mettre en jeu le pronostic vital maternel [4]. Le diagnostic est avant tout clinique et la symptomatologie est faite essentiellement de douleurs périnéales [5]. Vu la rareté de cette pathologie, sa prise en charge reste jusqu'à nos jours mal codifiée surtout pour les hématomes de grande taille. Ainsi chaque hématome péri-génital est singulier et le choix de la prise en charge est guidé par le bon sens clinique et les habitudes de chaque équipe [1].

La prise en charge des HP de petite taille (2]. Toutefois, devant des hématomes de gros volumes (>8cm) les pratiques divergent. En effet, dans ce cas, la plupart des auteurs préconisent systématiquement une prise en charge active qui repose sur la chirurgie et/ou l'embolisation [1]. D'autres équipes, dont la notre, optent plutôt pour une prise en charge conservatrice lorsque la patiente est stable sur les plans clinique et biologique [6, 7, 8]. Cette attitude est argumentée par le fait que l'hématome constitué exerce une pression sur les tissus qui l'entourent qui peut collaber le vaisseau qui saigne et permettre ainsi l'hémostase. Le fait d'évacuer cet hématome stable fait augmenter le risque de récidive du saignement [6, 7].

Nous partageons, dans cette situation, l'avis de plusieurs auteurs qui s'accordent pour dire que les indications thérapeutiques des HP dépendent essentiellement de la stabilité de l'état hémodynamique et biologique de la patiente, du caractère extensif ou non de l'hématome et des habitudes de l'équipe obstétricale et que la prise en compte uniquement de la taille de l'hématome isolée parait insuffisante pour poser une attitude thérapeutique [9, 10].

Nous avons proposé dans une publication précédente une attitude conservatrice pour les hématomes de tailles importantes (>8cm), stables, associés à une stabilité hémodynamique. Ces 3 cas vécus permettent d'étayer cette conduite [6]. Cette attitude nous parait très intéressante surtout pour les équipes qui ne disposent pas de possibilités d'embolisation radiologique. En effet, elle permet d'éviter une intervention chirurgicale (chirurgie du site lésionnel ou ligature des hypogastriques) souvent difficile vue les difficultés fréquentes de l'hémostase locale et dont certaines complications peuvent être redoutables (plaie urétrale, plaie veine iliaque interne ‘) [1]. Il faut rappeler aussi que l'embolisation artérielle reste un geste qui nécessite en plus d'un plateau technique approprié, des radiologues interventionnels expérimentés. En effet, même si les complications de cette technique sont rares, elles ne sont pas exceptionnelles (toxicité au produit de contraste, insuffisance rénale, nécrose utérine, ischémie sciatique crurale...) [11].

## Conclusion

L'hématome péri-génital est un évènement imprévisible, rare mais non exceptionnel auquel chaque obstétricien doit être préparé. Retenons que le choix du traitement dépend essentiellement du bilan lésionnel initial et de la rapidité de l'évolution de l'hématome. La prise en charge reste encore non consensuelle surtout pour les hématomes de gros volumes pour lesquels une prise en charge conservatrice parait être possible dans certaines conditions bien précises. D'autres études et publications à plus large effectif restent nécessaires pour pouvoir étayer cette conduite et espérer dresser un algorithme décisionnel qui puisse être le plus consensuel possible.
